# Supplementary material for: Modelling Skylarks (Alauda arvensis) to Predict Impacts of Changes in Land Management and Policy: Development and Testing of an Agent-Based Model
Source: PLoS One. 2013 Jun 6;8(6):e65803. doi: 10.1371/journal.pone.0065803 (PMC3675089; doi:10.1371/journal.pone.0065803)
Supplement: Supporting Information S4 — The skylark ODdox as a zipped archive. (ZIP) [file pone.0065803.s004.zip › Skylark_ODdox/pages.html]

ALMaSS Skylark ODdox: Related Pages


ALMaSS Skylark ODdox
 2.0


- Main Page
- Related Pages
- Classes
- Files

Related Pages

Here is a list of all related documentation pages:

|  |  |
| --- | --- |
| Crop Classes |  |
| Farm Management | Class for farm management |
| References |  |


- Generated on Thu Jan 10 2013 13:15:39 for ALMaSS Skylark ODdox by
   1.8.1.1
